# Characterization of a Dual Nonlinear Helmholtz Resonator

**DOI:** 10.3390/mi13112032

**Published:** 2022-11-20

**Authors:** Maher O. Al-Turk, Sajid Ali, Muhammad A. Hawwa

**Affiliations:** 1 Department of Mechanical Engineering & IRC for Advanced Materials, King Fahd University of Petroleum & Minerals, Dhahran 31261, Saudi Arabia; 2 Mechanical and Energy Engineering Department, Imam Abdulrahman Bin Faisal University, Dammam 31441, Saudi Arabia

**Keywords:** dual Helmholtz resonator, lumped-parameter analysis, nonlinear oscillations, bifurcation, phase portrait, hardening and softening

## Abstract

Resonant elements can generate small amounts of energy that make them pertinent for feeding miniaturized accelerometers with the energy needed. Suitable oscillator candidates are Helmholtz resonators, which have been, for a long time, analyzed and designed within the context of linear vibration. This study focuses on extracting nonlinear characteristics of a dual Helmholtz resonator (HR), with a neck-cavity–neck-cavity configuration, mounted on an acoustic waveguide with harmonically oscillating pressure. The mathematical model used for describing the resonator embraces inherent nonlinear air stiffness and the damping nonlinearity of hydrodynamic origin. Numerical solutions for the resonator’s nonlinear oscillations are obtained. Bifurcation diagrams are produced, indicating that the dual HR behaves in a deterministic fashion within the engineering practical limits. Phase portraits are drawn for the system, showing a quasi-periodic motion. Frequency response curves (FRC) are found to shift to the left at the lower resonant frequency indicating a softening behavior. FRC keep generally symmetric curves at the higher resonant frequency indicating a mostly linear behavior.

## 1. Introduction

Accelerometers are efficient devices for measuring the linear speed change of objects to which they are attached. Whether the object undergoes steady, transient, or oscillatory motion, accelerometers can measure accelerations associated with these motions. To make accelerometers more amenable to integration, they have been miniaturized by utilizing MEMS manufacturing processes. Thus, a proof mass motion causes a change in electrical potentials in the adjoining capacitive plates, indicating the occurrence of acceleration. Due to their key function in enhancing manufacturing processes, environmental sustainability, and human health, safety, and lifestyle, miniaturized accelerometers have been employed in smart instrumentation, seismic activity sensing, automotive, marine and aerospace navigation, and the wellbeing of living things. Although accelerometers are considered to be low-power devices, emerging applications (such as the Internet of Things-related functions, with repeated power-saving/standard operating modes) could trigger the need for micro- to milli-ampere power generation. The employment of MEMS accelerometers in an acoustic environment abound of resonating elements like Helmholtz resonators makes it relevant to utilize these resonators for small energy harvesting. In fact, over the past decade, Helmholtz resonators have been proposed for acoustic energy harvesting [1,2,3,4,5,6].

The Helmholtz resonator (HR) [7] started as a musical tuning idea that ended up at the heart of noise-control engineering. For more than a century, the HR concept has stimulated a considerable amount of research and development efforts. For the past decades, HRs have been utilized as efficient devices for selective noise reduction in automotive mufflers, aircraft turbofan engines, and HVAC waveguides.

In its simple form, a HR is made of an enclosed volume of gas connected to the “outside” by a small opening (known as a neck) including a mass of gas. As the gas mass in the neck is exposed to an outside pressure, it compresses the gas in the confined volume that reacts in an elastic manner, acting as a spring. Early analysis of HRs was based on assuming the air mass in the neck as a “slug” (i.e., solid object). Thus, the neck mass and the enclosed volume spring were modelled as a typical mass-spring system having a natural frequency [8]. The HR acts on eliminating any “outside” sound wave with a frequency that is equal to its natural frequency. Hence, the HR can act as a typical selective device for rejecting an undesired acoustic signal.

Researchers have been investigating HRs with enclosed volumes and necks having a wide variety of geometries, considering a purely linear elastic behavior of the confined air in the enclosed volumes. Relevant to the research reported in our paper is the work presented in [9,10,11,12,13] considering a dual HR which consisted of two HR connected in series, the invention of Hawwa [14] who put forward an adaptive dual HR, the works of Beck et al. [15] on dual resonance acoustic liners, and the analyses offered in [16] and [17] on periodic arrays comprised of dual Helmholtz resonators. The importance of introducing dual HRs stems from their capabilities of controlling hybrid noise at low frequencies, below 1000 Hz.

When researchers started to consider the associated sound field–fluid flow problem within the Helmholtz resonator, they started to pay attention to the damping due to friction resistance, jet effect, and the nonlinear nature of air stiffness. Based on hints made in references [18] and [19] and experimental findings reported in reference [20], Zinn [21] presented a resonator’s flow field with external pressure oscillations, focusing on the effect of nonlinear resistance. Sirignano [22] considered the nonlinear damping of pressure oscillations in a HR determining the admittance, resistance, and reactance. Hersh and Walker [23,24] calculated the acoustic impedance of Helmholtz resonators using a semi-empirical model in the form of a non-linear governing equation. Innes and Crighton [25] presented a matched asymptotic expansion solution of the model proposed in [23,24]. Boullosa and Orduña-Bustamante [26] used the thermodynamic process occurring in the air inside the enclosed volume of the HR to study its elastic nonlinear behavior and made measurements that quantitatively confirmed the predictions from their model. The performance of dual HRs connected in series was presented in [9]. Lumped approach was applied for the resonance frequencies and transmission loss of the 2DOF configuration.

Hersh et al. [27] used physically inspired modelling assumptions to find nonlinear corrections of the impedance gotten in earlier studies. Yu et al. [28] obtained the nonlinear amplitude-frequency response of an acoustic Helmholtz resonator, giving an explanation of the downward shift of resonance frequency. Singh and Rienstra [29] considered nonlinear effects in HRs that stem from hydrodynamic sources, including vortex shedding at the outflow from the opening. Achilleos et al. [30] used a transmission-line approach to realize a nonlinear dynamic model, which led to obtaining acoustic soliton solutions for the pressure in a waveguide connected to HRs. Softening and hardening behaviors for a high-amplitude nonlinear HR involving nonlinear restoring and damping forces is reported in [31,32,33]. A compliant wall was introduced around the acoustic cavity in [32]. The dynamics response of the HR and the effectiveness of the wall in terms of sound observation were investigated. Vargas et al. [31] have considered HRs with non-linear restoring. The results obtained exhibit non-linear hardening and softening behaviors.

To the best of authors’ knowledge, nonlinear analysis and/or design of dual Helmholtz resonators has not yet been considered. Accounting for nonlinearities of both stiffness and damping could lead to improved accuracy of the resonator’s response. The aim of this paper is to investigate nonlinear characteristics of a dual Helmholtz resonator (HR), with a neck-cavity–neck-cavity configuration, attached to an acoustic waveguide having harmonically oscillating pressure. The equations of motion describing the resonator are solved numerically to obtain bifurcation plots, phase portrait diagrams, and frequency response curves. These results would help in determining (i) the frequency domain(s) over which the resonator’s response is deterministic/chaotic, (ii) the displacement-velocity relationship for identifying trajectories followed by the resonator as a dynamical system, (iii) and categorizing any hardening/softening performances. These characterizations can be utilized for designing acoustic filters that can be utilized for harvesting energy that can feed miniaturized accelerometers.

## 2. Mathematical Formulation

**Figure 1:**
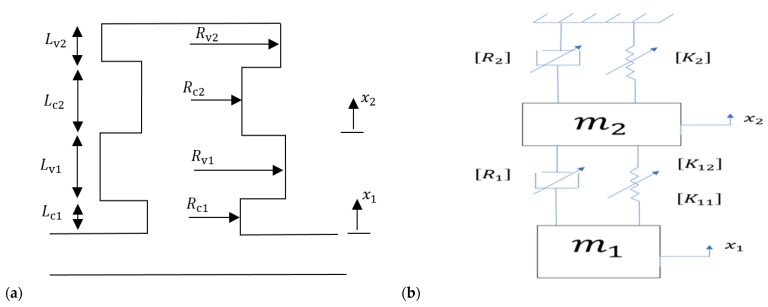
(**a**) The dual nonlinear HR, and (**b**) its equivalent lumped-parameter system.

Shown in Figure 1a is a dual Helmholtz resonator, consisting of two necks and two cavities, and mounted at the side of an acoustic waveguide. The dual HR is idealized in Figure 1b as a two-degree-of-freedom lumped-parameter system that can mathematically be described by the following governing equations

(1) $$m_{1}{ẍ}_{c1}+F_{damping}+F_{elasticity\;}=-F_{duct}$$

(2) $$m_{2}{ẍ}_{c2}-F_{damping}-F_{elasticity\;}=0$$

where $m_{1,2}$ is the mass of air inside the first and second neck, respectively, $x_{c1,2}$ is the displacement of the air of the neck of the first and second HRs, respectively, and the double dots indicate differentiation with respect to time. The forces, $F_{damping}\;\mathrm{and}\;F_{elasticity\;}$ are both nonlinear functions of velocities and displacements, respectively, systems with nonlinear terms of stiffness and damping. $F_{duct}$ is the force applied to the dual HR as a result to pressure fluctuations in the acoustic waveguide.

Following [28] and [31], the air inside each cavity (volume) is assumed to behave nonlinearly according to the following relationship:

(3) $$\Delta p_{j}=-\rho_{0}L_{ej}\omega_{0j}{}^{2}\left(x_{cj}-\frac{\left(\gamma +1\right)A_{cj}}{2V_{j}}x_{cj}^{2}+\frac{\left(\gamma +1\right)\left(\gamma +2\right)A_{cj}^{2}}{6V_{j}^{2}}x_{cj}^{3}\right)$$

where the subscript *j* (*j* = 1, 2) indicates the *jth* resonator, $\Delta p_{j}$ is the pressure change inside the cavity, $\rho_{0}$ is the air density, $L_{ej}$ is the effective length of the neck ($L_{ej}=L_{cj}+\frac{16R_{cj}}{3\pi }$), where$\;L_{cj}$ is the neck length and $R_{cj}$ is the radius of the cylindrical neck, $\omega_{0j}$ is the resonator’s natural frequency, $x_{cj}$ is the displacement of the air on the neck of the first HHR, γ is specific heat ratio, $A_{cj}$ is the cross-sectional area of the neck, and $V_{j}$ is the volume of the cavity. The linear damping and nonlinear damping due to the jet phenomenon [30] can be written as

(4) $$D_{j}=\frac{2m_{j}A_{cj}}{2\rho_{0}L_{ej}}R\left(Z_{in}+Z_{vis}\right)\;\left(\frac{dx_{cj}}{dt}\right)+\frac{\zeta m_{j}}{2L_{ej}}\left(\frac{dx_{cj}}{dt}\right)\left(\frac{dx_{cj}}{dt}\right)$$

where $D_{j}$ is the damping inside the neck, $m_{j}$ is the equivalent mass of air inside the neck, $Z_{in}$ is the acoustic impedance, $Z_{vis}$ is the frictional acoustic impedance, ζ is the coefficient of the total hydraulic resistance.

In the sequel, the effectiveness of the dual HR is assessed by studying the dynamic behavior of its equivalent spring-damper-mass system. Thus, the governing equation of motion for each HR is derived using Newton’s second law of motion. Therefore, the rate of change of the linear momentum equation for the two masses of air inside the necks are

(5) $$\begin{array}{l} m_{1}\frac{d^{2}x_{c1}}{dt^{2}}+\frac{2\;m_{1}A_{c1}}{2\rho_{0}L_{e1}}R\left(Z_{in}+Z_{vis}\right)\left(\frac{dx_{c1}}{dt}-\frac{dx_{c2}}{dt}\right)+\frac{\zeta m_{1}}{2L_{e1}}\left(\frac{dx_{c1}}{dt}\frac{dx_{c1}}{dt}-\frac{dx_{c2}}{dt}\frac{dx_{c2}}{dt}\right)\\ +\frac{\rho_{0}c_{0}^{2}A_{c1}}{V_{1}}(A_{c1}x_{c1}-A_{c2}x_{c2}-\frac{\left(\gamma +1\right)}{2V_{1}}\left(A_{c1}^{2}x_{c1}^{2}-A_{c2}^{2}x_{c2}^{2}\right)\\ +\frac{\left(\gamma +1)(\gamma +2\right)}{6V_{1}^{2}}\left(A_{c1}^{3}x_{c1}^{3}-A_{c2}^{3}x_{c2}^{3}\right))=-p\;A_{c1} \end{array}$$

(6) $$\begin{array}{l} m_{2}\frac{d^{2}x_{c2}}{dt^{2}}+\frac{2m_{1}A_{c1}}{2\rho_{0}L_{e1}}R\left(Z_{in}+Z_{vis}\right)\left(\frac{dx_{c2}}{dt}-\frac{dx_{c1}}{dt}\right)\\ +\frac{\zeta m_{1}}{2L_{e1}}\left(\frac{dx_{c2}}{dt}\frac{dx_{c2}}{dt}-\frac{dx_{c1}}{dt}\frac{dx_{c1}}{dt}\right)\\ +\frac{\rho_{0}c_{0}^{2}A_{c2}}{V_{1}}(A_{c2}x_{c2}-A_{c1}x_{c1}-\frac{\left(\gamma +1\right)}{2V_{1}}\left(A_{c2}^{2}x_{c2}^{2}-A_{c1}^{2}x_{c1}^{2}\right)\\ +\frac{\left(\gamma +1)(\gamma +2\right)}{6V_{1}^{2}}\left(A_{c2}^{3}x_{c2}^{3}-A_{c1}^{3}x_{c1}^{3}\right))+\frac{2m_{2}A_{c2}}{2\rho_{0}L_{e2}}R\left(Z_{in}+Z_{vis}\right)\left(\frac{dx_{c2}}{dt}\right)\\ +\frac{\zeta m_{2}}{2L_{e2}}\left(\frac{dx_{c2}}{dt}\right)\left(\frac{dx_{c2}}{dt}\right)\\ +\frac{\rho_{0}c_{0}^{2}A_{c2}^{2}}{V_{2}}\left(x_{c2}-\frac{(\gamma +1)A_{c2}}{2V_{2}}x_{c2}^{2}+\frac{(\gamma +1)(\gamma +2)A_{c2}^{2}}{6V_{2}^{2}}x_{c2}^{3}\right)=0 \end{array}$$

Note that the dual HR is forced into oscillatory motion by the pressure fluctuation, p, in the acoustic waveguide. In order to facilitate the dynamic analysis, let us introduce the dimensionless variables: t* =$\;\omega_{01}t$, $x_{1}$=$\;\frac{x_{c1}A_{c1}}{V_{1}}$,$\;x_{2}$=$\;\frac{x_{c2}A_{c2}}{V_{2}}$ as dimensionless time, displacement of the first mass, the displacement of the second mass, respectively. In order to facilitate the analysis, let us define the following parameters:

(i) The ratio of the masses of air inside the two necks: $r_{1}=\frac{m_{1}}{m_{2}}.$

(ii) The ratio of the natural frequencies of the first and second HR: $r_{2}=\frac{\omega_{02}}{\omega_{01}}$ where $\omega_{01}=\sqrt{\frac{c_{0}^{2}A_{c1}}{L_{e1}V_{1}}},\;\omega_{02}=\sqrt{\frac{c_{0}^{2}A_{c2}}{L_{e2}V_{2}}}$ and $c_{0}$ is the speed of sound.

(iii) The ratio of the forcing frequency of pressure fluctuation and the natural frequency of the first HR: $\Omega =\frac{\omega }{\omega_{01}}$.

(iv) The stiffness coefficients: $k_{1}=-\alpha =-\frac{\left(\gamma +1\right)}{2}$ and $k_{2}=\beta =\frac{\left(\gamma +1\right)\left(\gamma +2\right)}{6}$.

(v) The damping coefficients: $c_{1}=\frac{\delta_{1}}{\omega_{01}}=\frac{2A_{c1}}{2\rho_{0}L_{e1}\omega_{01}}R\left(Z_{in}+Z_{vis}\right),\;c_{2}=\frac{\delta_{2}}{\omega_{01}}=\frac{2A_{c2}}{2\rho_{0}L_{e2}\omega_{01}}R\left(Z_{in}+Z_{vis}\right),$ $c_{3}=\frac{\zeta V_{1}}{2L_{e1}A_{c1}},$ $c_{4}=\frac{\zeta V_{2}}{2L_{e2}A_{c2}\;}$.

(vi) The non-dimensional pressure amplitude: $P=\frac{-pA_{c1}}{\rho_{0}L_{e1}\omega_{01}^{2}V_{1}}$.

Then, the following normalized governing equations of motion are obtained:

(7) $$\begin{array}{l} \frac{d^{2}x_{1}}{\;dt^{*2}}+c_{1}\left(\frac{dx_{1}}{dt^{*}}-\frac{A_{c1}V_{2}}{A_{c2}V_{1}}\frac{dx_{2}}{dt^{*}}\right)+c_{3}\left(\frac{dx_{1}}{dt^{*}}\left|\frac{dx_{1}}{dt^{*}}\right|-\frac{A_{c1}^{2}V_{2}^{2}}{A_{c2}^{2}V_{1}^{2}}\frac{dx_{2}}{dt^{*}}\left|\frac{dx_{2}}{dt^{*}}\right|\right)\\ +\left(x_{1}-\frac{V_{2}}{V_{1}}x_{2}\right)+k_{1}\left(x_{1}^{2}-\frac{V_{2}^{2}}{V_{1}^{2}}x_{2}^{2}\right)+k_{2}\left(x_{1}^{3}-\frac{V_{2}^{3}}{V_{1}^{3}}x_{2}^{3}\right)=Pcos\left(\Omega t^{*}\right) \end{array}$$

(8) $$\begin{array}{l} \frac{d^{2}x_{2}}{\;dt^{*2}}+r_{1}c_{1}\left(\frac{dx_{2}}{dt^{*}}-\frac{A_{c2}V_{1}}{A_{c1}V_{2}}\frac{dx_{1}}{dt^{*}}\right)\\ +r_{1}c_{3}\frac{A_{c1}}{V_{1}}\left(\frac{V_{2}}{A_{c2}}\frac{dx_{2}}{dt^{*}}\left|\frac{dx_{2}}{dt^{*}}\right|-\frac{A_{c2}V_{1}^{2}}{A_{c1}^{2}V_{2}}\frac{dx_{1}}{dt^{*}}\left|\frac{dx_{1}}{dt^{*}}\right|\right)\\ +\frac{L_{c1}^{\prime }}{L_{c2}^{\prime }A_{c1}}\left(A_{c2}x_{2}-\frac{A_{c2}V_{1}}{V_{2}}x_{1}\right)-\frac{L_{c1}^{\prime }k_{1}}{\;L_{c2}^{\prime }A_{c1}V_{1}}\left(V_{2}A_{c2}x_{2}^{2}-\frac{A_{c2}V_{1}^{2}}{V_{2}}x_{1}^{2}\right)\\ +\frac{L_{c1}^{\prime }k_{2}}{\;L_{c2}^{\prime }A_{c1}V_{1}^{2}}\left(A_{c2}V_{2}^{2}x_{2}^{3}-\frac{A_{c2}V_{1}^{3}}{V_{2}}x_{1}^{3}\right)+c_{2}\frac{dx_{2}}{dt^{*}}+c_{4}\frac{dx_{2}}{dt^{*}}\left|\frac{dx_{2}}{dt^{*}}\right|+r_{2}^{2}x_{2}\\ -k_{1}r_{2}^{2}x_{2}^{2}+k_{2}r_{2}^{2}x_{2}^{3}=0 \end{array}$$

Equations (7) and (8) represent two coupled inhomogeneous differential equations, involving quadratic and cubic nonlinearities. It is worth noting that the linear version of Equations (7) and (8) represents the case of a linear dual HR, which was considered by Xu et al. [9] with eliminating the damping term. Equation (7) represents the case of a single nonlinear HR, which was considered by Vargas et al. [31]. For building confidence in the developed model, the works in [9] and [31] are in fact obtained as special cases of the model considered in this paper.

## 3. Results and Discussions

A numerical solution of the coupled Equations (7) and (8) is sought using the MATLAB code *ode45*, which is based on an explicit Runge–Kutta method. With zero initial deflection and velocity, the equations are integrated over the period of dimensionless time t* = [0–20,000] to obtain a steady-state response. The physical parameters of the fluid contained in the dual HR that are used in calculations are listed in Table 1.

Two representative cases of dual nonlinear HRs, connected to an acoustic waveguide, are considered: (i) The first dual HR is constructed of two identical single HRs. (ii) The second dual HR is constructed of two single HRs that have a different volume than that of the other HR, as clarified in Table 2.

The dual HR is constructed on an acoustic waveguide for the purpose of noise control and the possibility of small energy generation. The pressure fluctuations in the acoustic waveguide are considered to vary such that the pressure amplitude (P) corresponding to a sound pressure level within (90~170) dB.

In order to evaluate the nonlinear dual HR performance, let us first consider the bifurcation diagram with the sound pressure level in the acoustic waveguide as a bifurcation parameter.

### 3.1. Bifurcation Diagram

**Figure 2:**
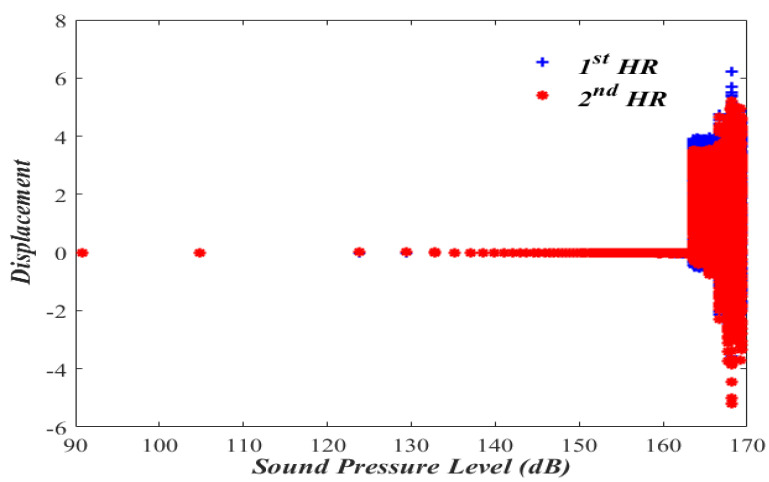
Bifurcation diagram for a nonlinear dual HR (Case I). Note that the 1st and the 2nd HRs have the same displacement before the chaotic response.

**Figure 3:**
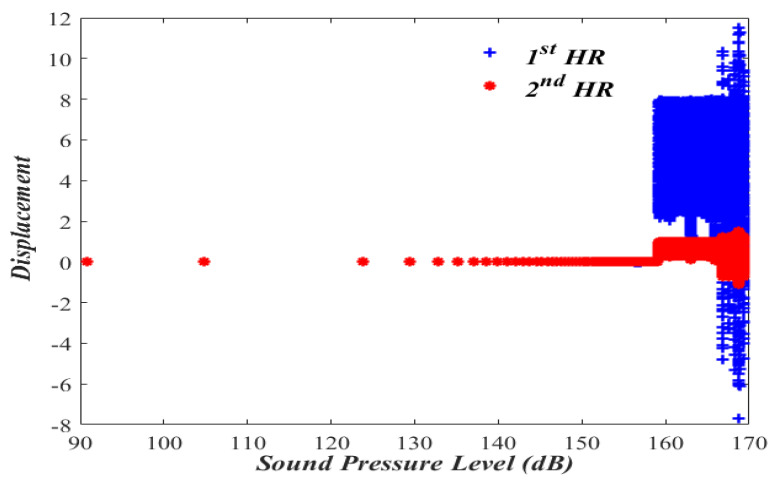
Bifurcation diagram for a nonlinear dual HR (Case II). Note that the 1st and the 2nd HRs have the same displacement before the chaotic response.

The importance of bifurcation diagram stems from the fact that they help to determine if the nonlinear system behaves in a deterministic or a chaotic manner. The bifurcation diagram is obtained by calculating $x_{1}$ and $x_{2}$ while varying the amplitude of the forcing pressure (P) inside the acoustic waveguide and keeping the nondimensional forcing frequency, Ω, at 1.01, as shown in Figure 2 and Figure 3.

Figure 2 shows that the periodic oscillatory behaviors of $x_{1}$ and $x_{2}$ of Case I over the range of sound pressure level of (90–140) dB. A nonlinear jump phenomenon followed by the chaotic response is observed to take place at 163 dB, which is beyond most practical engineering applications. A similar oscillatory behavior is also observed in Figure 3 of Case II, where a nonlinear jump phenomenon followed by a chaotic response is observed to take place a little earlier, at 159 dB.

To study the periodic response of the dual nonlinear HR, two types of results are obtained; the phase portrait and frequency response.

### 3.2. Frequency Response Curves

**Figure 4:**
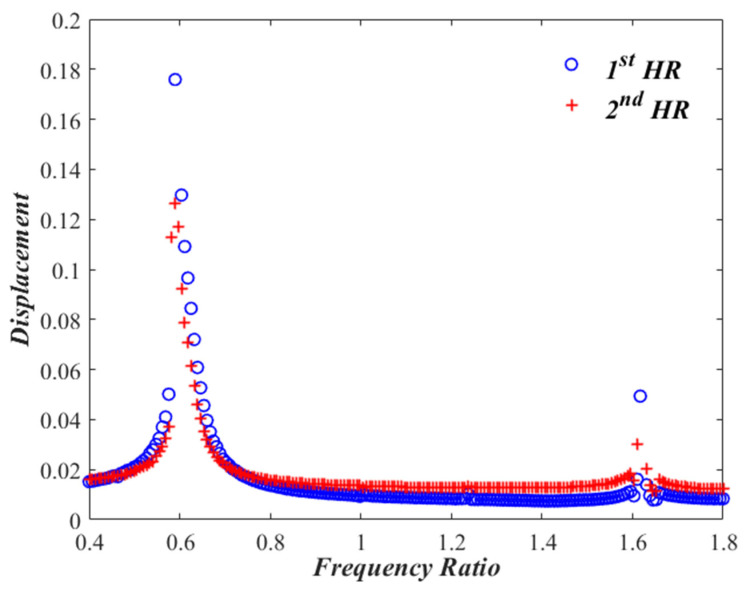
Frequency response curves for $x_{1,2\;}$of the nonlinear dual HR (Case I).

**Figure 5:**
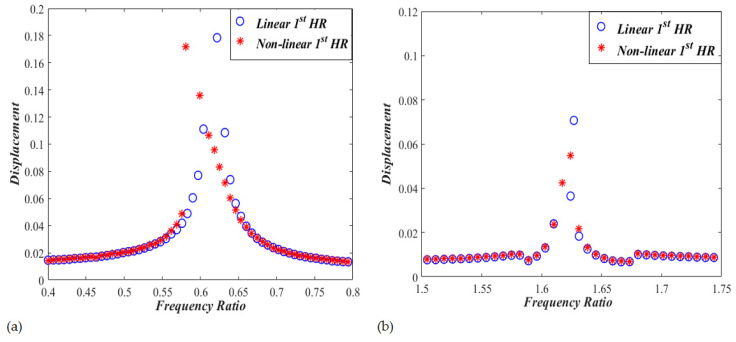
Frequency response curves for linear and non-linear nonlinear dual HRs (CASE I). (**a**) first resonant response, and (**b**) second resonant response.

Let us now monitor the variation of $x_{1}$ and $x_{2}\;$with changing the excitation frequency, Ω, from 0.4 to 1.8, while keeping the sound pressure level at 150 dB. Performing a frequency sweep and solving Equations (5) and (6) representing Case I several times, one gets response curves shown in Figure 4 which exhibit two resonances at the frequency ratios of Ω= 0.60 and Ω=1.62. The nonlinear effect of the dual HR is manifested by a softening behavior at the first resonant frequency, with amplitudes of $x_{1}\approx 0.18$ and $x_{2}\approx 0.125$ At the second resonant frequency, however, a predominantly linear behavior is noted, with amplitudes of $x_{1}\approx 0.054$ and $x_{2}\approx 0.025$. In order to have a clear visualization of the detected softening effect, the frequency response for the linear problem for Case I is obtained after removing nonlinear terms from the model, and compared to that of the non-linear model. Zoomed-in pictures are demonstrated in Figure 5 for the frequency response around the two resonant frequencies. While the linear curves have straight-up peaks, the nonlinear response around the first resonant frequency bends towards the left indicating softening effect. However, the nonlinear response around the second resonant frequency is very much behaving like the linear response.

**Figure 6:**
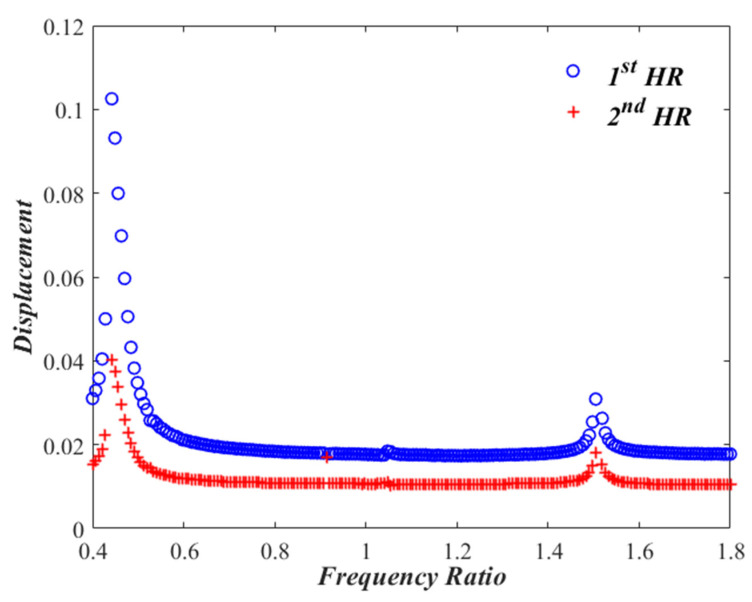
Frequency response curves for $x_{1,2\;}$of the nonlinear dual HR (Case II).

**Figure 7:**
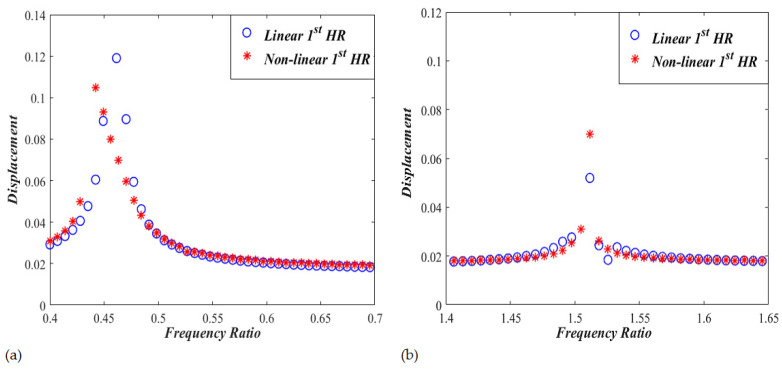
Frequency response curves for linear and non-linear nonlinear dual HRs (CASE II). (**a**) first resonant response, and (**b**) second resonant response.

The frequency response curves are also obtained for Case II by changing the excitation frequency, Ω, from 0.4 to 1.8, while keeping the sound pressure level of 150 dB. Figure 6 shows two resonant frequencies occurring at the frequency ratios of Ω= 0.45 and Ω=1.54. It is observed that a slight softening behavior exists at the first resonant frequency, with amplitudes of $x_{1}\approx 0.102$ and $x_{2}\approx 0.041$. The second resonant frequency is slightly nonlinear with amplitudes of $x_{1}\approx 0.033$ and $x_{2}\approx 0.021$. Zoomed-in pictures comparing linear and nonlinear resonant frequency responses are shown in Figure 7. The nonlinear response around the first resonant frequency bends towards the left indicating softening effect. The nonlinear response around the second resonant frequency does not lean towards the left or right direction, thus, demonstrating a response that is pretty much linear.

### 3.3. Phase Portrait

Phase portrait is an interesting geometric demonstration of the trajectories followed by a dynamical system and a valuable tool for identifying stability regions.

**Figure 8:**
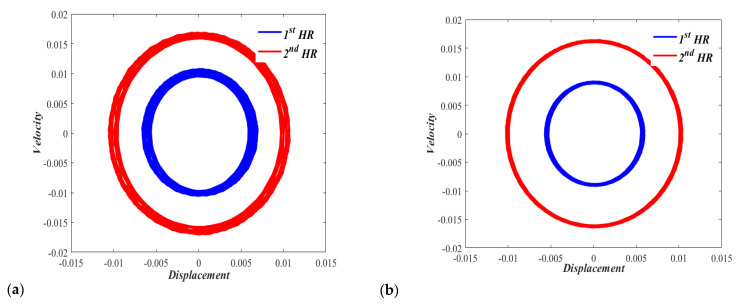
Phase portrait for the nonlinear dual HR excited by p = 150 dB, (**a**) Ω = 0.65, and (**b**) Ω = 1.65.

On a phase plane showing the displacement-velocity relationship for Case I with zero displacements and zero velocities initial conditions, Figure 8a shows a slightly off periodic motion (which is represented by a circular-shaped response) under the excitation $F_{duct}=P\cos\left(0.65t*\right)$, where *P* is considered equivalent to a sound pressure level of 150 dB. Figure 8b also shows a neutrally stable phase plane portrait with a similar quasiperiodic motion under the excitation $F_{duct}=P\cos\left(1.65t*\right)$.

**Figure 9:**
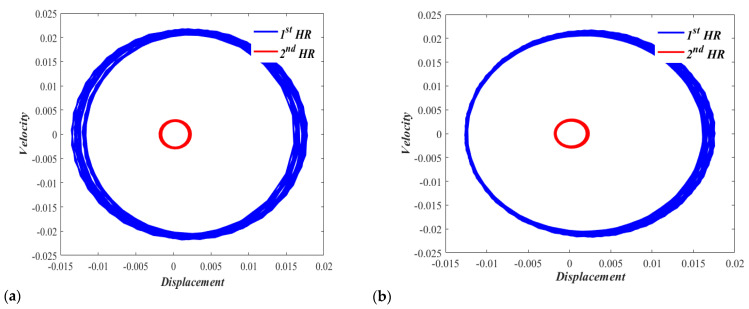
Phase portrait for the nonlinear dual HR excited by p = 150 dB, (**a**) Ω = 0.47, and (**b**) Ω = 1.57.

Similarly, the phase plane obtained for Case II with zero displacement and zero velocity initial conditions is obtained. Figure 9a shows a clearly off-periodic motion with an equilibrium point slightly shifted to the right under the excitation $F_{duct}=P\cos\left(0.47t*\right)$, where *P* is considered equivalent to a sound pressure level of 150 dB. Figure 9b shows a neutrally stable phase plane portrait with off-periodic motion and an equilibrium point noticeably shifted to the right under the excitation $F_{duct}=P\cos\left(1.57t*\right)$.

**Figure 10:**
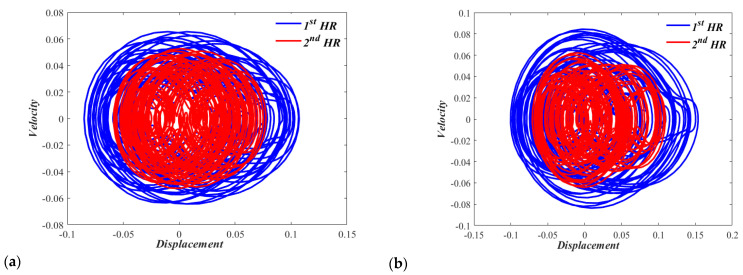
Phase portrait for the nonlinear (CASE I) dual HR excited by, (**a**) p = 165 dB, and (**a**) p = 170 dB.

**Figure 11:**
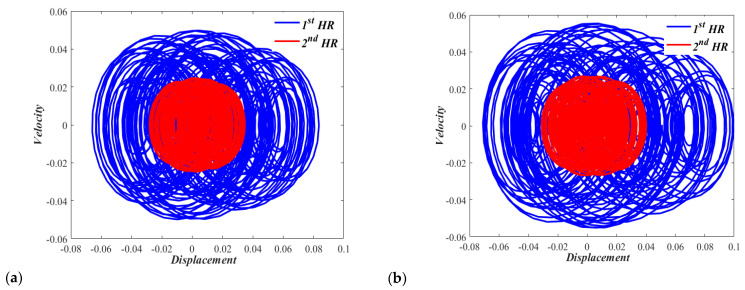
Phase portrait for the nonlinear (CASE II) dual HR excited by, (**a**) p = 165 dB, and (**b**) p = 170 dB.

It would also be interesting to consider phase portraits at higher sound pressure levels to examine the chaotic behavior of the nonlinear dual HR with zero displacements and zero velocities initial conditions. Figure 10a,b show the chaotic behaviors of Case I dual resonator at sound pressure levels of 165 dB and 170 dB, respectively. Figure 11a,b show the chaotic behaviors of the Case II dual resonator at sound pressure levels of 165 dB and 170 dB, respectively. The irregular and unpredictable nature is clear in all of these figures. In none of these figures, any individual HR of nonlinear dual HR repeats its past history.

## 4. Conclusions

An analysis was made to examine the nonlinear characteristics of a dual Helmholtz resonator attached to an acoustic waveguide with a harmonically oscillating pressure. The mathematical model describing the resonator comprised two coupled inhomogeneous differential equations involving quadratic and cubic nonlinearities. The numerical solution indicated that the response of the dual HR was non-chaotic over the majority of the practical forcing frequency spectrum inside an acoustic waveguide. The response, however, involved a nonlinear jump phenomenon followed by a chaotic response at an oscillating acoustic pressure with an amplitude of 158 dB. Frequency response curves showed a softening behavior at the first resonant frequency and a chiefly linear behavior at the second resonant frequency. Phase portrait diagrams in the displacement-velocity plane showed quasiperiodic motion characteristics. The aim of the study was to direct the attention towards considering nonlinear effects when analyzing and designing Helmholtz resonators, which could be ideal energy harvesters for low-power applications, such as miniaturized accelerometers.

## Figures and Tables

**Table 1:** Physical Parameters of the dual nonlinear HR.

Parameter	Value
Linear Damping Coefficient ($\delta_{j})$	8.67 s^−1^
Speed of Sound ($c_{0})$	340.0 m/s
Density of Air ($\rho_{0}$)	1.2 kg/m^3^
Specific Heat Ratio (γ)	1.4
Coefficient of Hydraulic Resistance (ζ)	2.1 × 10^−4^

**Table 2:** The construction of the small-size dual nonlinear HR.

	Case I (Identical HRs)		Case II (Different HRs)	
Parameter	1st HR	2nd HR	1st HR	2nd HR
Radius of the Neck ($R_{cj}$)	0.1 mm	0.1 mm	0.1 mm	0.1 mm
Length of the Neck ($L_{cj}$)	0.2 mm	0.2 mm	0.2 mm	0.2 mm
Mass of air in the neck $\left(m_{j}\right)$	398.6 × 10^−9^ kg	398.6 × 10^−9^ kg	398.6 × 10^−9^ kg	398.6 × 10^−9^ kg
Radius of the Cavity ($R_{Vj}$)	0.8 mm	0.8 mm	0.4 mm	0.8 mm
Length of the Cavity ($L_{Vj}$)	0.6 mm	0.6 mm	0.3 mm	0.6 mm
Volume of the Cavity $\left(V_{j}\right)$	1.206 mm^3^	1.206 mm^3^	0.150 mm^3^	1.206 mm^3^
